# The Function of FGFR1 Signalling in the Spinal Cord: Therapeutic Approaches Using FGFR1 Ligands after Spinal Cord Injury

**DOI:** 10.1155/2017/2740768

**Published:** 2017-01-18

**Authors:** Barbara Haenzi, Lawrence D. F. Moon

**Affiliations:** Neurorestoration Group, Wolfson Centre for Age-Related Diseases, King's College London, London SE1 1UL, UK

## Abstract

Extensive research is ongoing that concentrates on finding therapies to enhance CNS regeneration after spinal cord injury (SCI) and to cure paralysis. This review sheds light on the role of the FGFR pathway in the injured spinal cord and discusses various therapies that use FGFR activating ligands to promote regeneration after SCI. We discuss studies that use peripheral nerve grafts or Schwann cell grafts in combination with FGF1 or FGF2 supplementation. Most of these studies show evidence that these therapies successfully enhance axon regeneration into the graft. Further they provide evidence for partial recovery of sensory function shown by electrophysiology and motor activity evidenced by behavioural data. We also present one study that indicates that combination with additional, synergistic factors might further drive the system towards functional regeneration. In essence, this review summarises the potential of nerve and cell grafts combined with FGF1/2 supplementation to improve outcome even after severe spinal cord injury.

## 1. Introduction

### 1.1. Spinal Cord Injury

Spinal cord injury (SCI) is a severe condition with an annual incidence of 1000 people each year in the UK and Ireland. This results in high costs that are currently at £1 billion per annum in the UK and Ireland (http://www.spinal-research.org/research-matters/spinal-cord-injury/facts-and-figures/). While there is good regeneration of peripheral nerves, injury to the central nervous system (CNS) is permanent since injured CNS axons do not regenerate long distances back to their original targets. Nonetheless, there is a certain degree of spontaneous repair, for example, via differentiation of precursor cells, axon sprouting, and building of new spinal circuits [1, 2]. These are areas that can be targeted by research in order to find new therapeutic approaches to increase axon regeneration after damage to the CNS. This review will concentrate on SCI and the function of the fibroblast growth factor receptor (FGFR) pathway in regeneration of injured axons.

It has been accepted that the devastating consequences of SCI are due to the limited capacity of lesioned CNS axons to undergo morphological and functional recovery, loss of neurons in the epicentre [3], and a conduction block of spared axons due to demyelination [4]. Causative factors for the inability of CNS axons to regenerate are a combination of factors including intrinsic and extrinsic factors. Intrinsic factors include progrowth genes that are not expressed by mature, injured neurons such as GAP-43 [5] and KLF7 [6] or antigrowth genes that are expressed by mature, injured neurons such as KLF4 [7]. Extrinsic factors are, for instance, lack of trophic support and the presence of inhibitory glial influences in the local environment, as reviewed by several groups [8–11].

SCI can be caused by contusion, compression, penetration, or maceration. All injuries cause massive damage to the spinal cord and induce a cascade of events. The immediate reaction consists of axotomy, haemorrhage, and ischema and then apoptosis and necrosis of cells including neurons, oligodendrocytes, and astrocytes. Secondary effects are further apoptosis, demyelination of axons, and the invasion of immune cells, such as macrophages, neutrophils, and T cells and activation of microglia [12–14]. Subsequently, a glial scar is formed at the injury site, which consists of reactive astrocytes, glial progenitors, microglia, macrophages [15, 16], fibroblasts, and Schwann cells [17, 18]. Importantly, numerous regeneration inhibitory molecules are found in the scar, such a Nogo-A and chondroitin sulphate proteoglycans (CSPGs) [19–21]. However, even though for many years the glial scar has been believed to have detrimental effects on axon regeneration, there is more and more evidence emerging that show that there is also a beneficial effect of the glial scar on axon regeneration [22]. The primary injury is immediate and irreversible, but the secondary injury evolves over time and provides a window of opportunity for treatment.

There has been much focus of research on how to promote regeneration of injured axons. This review focuses on therapies that manipulate the FGFR pathway to promote recovery after SCI.

### 1.2. The FGFR Pathway

**Figure 1:**
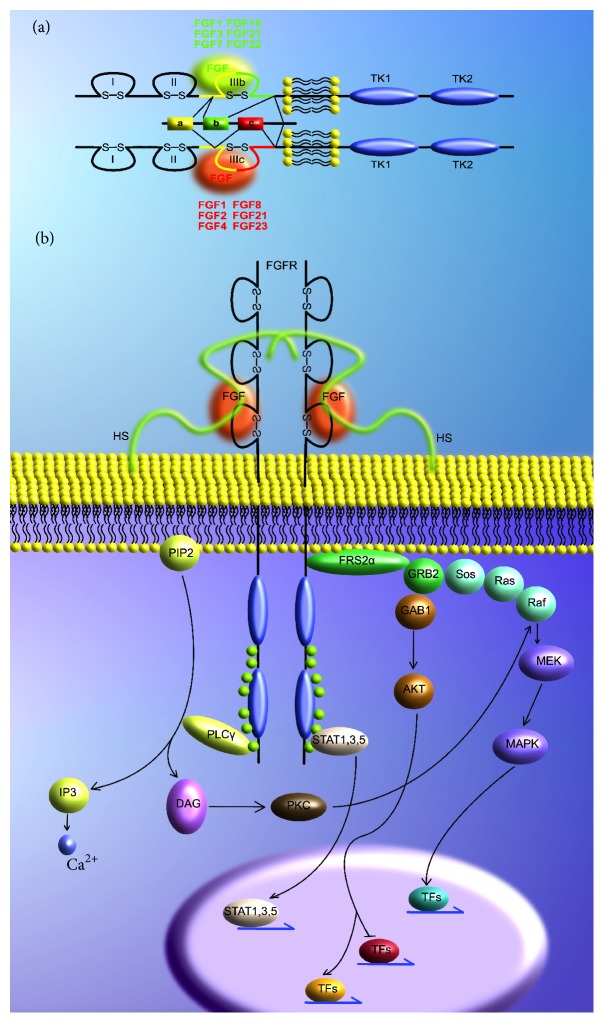
The FGFR signaling pathway. (a) Schematic of the structure of FGF receptors and the binding specificities of the FGF ligands. (b) Depiction of the FGFR downstream signalling. HS: heparan sulfate; PIP2: phosphatidylinositol 4,5-bisphosphate; IP3 inositoltriphosphate; PLC*γ*: phospholipase C; DAG: diacylglycerol; GRB2: growth factor receptor-bound protein 2; GAB1: GRB2 associated binding protein 1; Sos: son of sevenless; MEK: MAPK/ERK kinase; MAPK: mitogen activated protein kinase; STAT: signal transducer and activator of transcription; TFs: transcription factors.

The FGFR pathway is important in development, maintenance, and regeneration of the nervous system. The FGFR superfamily consists of 4 different receptors FGFR1–4. FGFR1–3 are each found in two different isoforms, named b and c, while FGFR4 exists only in the c isoform [23] (Figure 1(a)). The predominant receptors in the CNS are FGFR1 and 2. To date, 22 different FGF ligands have been identified, whereby FGF1 and FGF2 bind all 4 receptors. They are both secreted proteins and signal in a para- or autocrine fashion. Downstream of FGFR1 activation is three mayor pathways: AKT- and ERK-pathway that are activated via Fibroblast Growth Factor Receptor Substrate 2 (FRS2) and PLC*γ* which is FRS2 independent [23] (Figure 1(b)). Many therapeutic approaches aim at increasing the concentration of either of these two ligands at the injury site and this will be discussed in more detail below.

Further to the 22 FGF ligands NCAM, N-cadherin, and L1 have been shown to activate the FGFR pathway in the nervous system [24–26]. Binding of these ligands to the FGFR1 leads to binding and activation of PLC*γ* and subsequently to the production of diacylglycerol and an increase in cytosolic Ca^2+^ [27]. Diacylglycerol then produces ligands that activate the endocannabinoid receptor [28]. Two endocannabinoid receptors exist; the endocannabinoid receptor 1 (CB1) is mainly expressed in the CNS [29]. CB1 is a 7 transmembrane receptor [29–32] that can be activated by anandamide and 2-arachidonylglycerol [33, 34]. Activation of CB1 leads to activation of Ca^2+^ and K^+^ channels, the PI3K pathway, and various MAPK pathways, such as ERK, p38, and the JNK pathway. It also leads to inhibition of cAMP production and PKA activity. It has been shown that activation of the JNK pathway downstream of CB1 activation converges into STAT3 activation which leads to neurite outgrowth [35, 36]. Manipulating the endocannabinoid system downstream of the FGFR pathway is one other important line of therapeutic research to enhance regeneration in the CNS.

**Figure 2:**
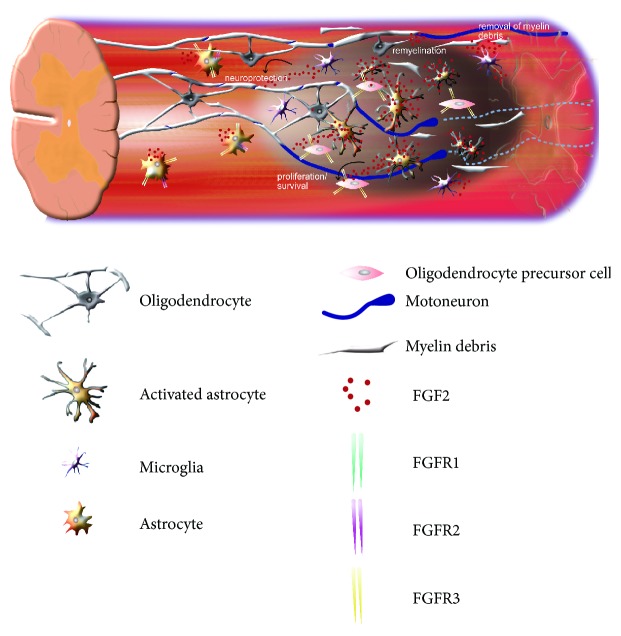
Overview of the spinal cord lesion and cells found in the lesion with their action stimulated by FGFR signaling.

A summary of the functions of FGFR signalling at the lesion site can be found in Figure 2.

## 2. Expression of FGF Ligands and Receptors in the Nervous System

### 2.1. Myelinating Cells

SCI causes acute and chronic loss of oligodendrocytes for up to a year after injury in rats and monkeys [37, 38]. Remyelination of spared neurons would enhance their ability to conduct action potentials efficiently. How this happens spontaneously is currently under debate. There is evidence for two mechanisms: (1) Schwann cells migrate from the periphery to the site of injury [39–41] or (2) oligodendrocyte precursor cells (OPCs) differentiate into mature myelinating cells [42, 43]. Indeed, it is also possible that both mechanisms act synergistically. It has been shown that FGF2 in concert with PDGF-A, IGF-I, and TGF-*β*1 plays a role in remyelination after lysolecithin induced demyelination of neurons [44]. Oligodendrocytes and their progenitors express FGF2 and FGFR1, 2, or 3 depending on their state of progression through their lineage. FGFR1 expression increases as the lineage progresses, FGFR3 is most highly expressed in late progenitors and then declines, and FGF2 is expressed in terminally differentiated oligodendrocytes [45]. Further, oligodendrocytes are dependent on FGF2 for differentiation and proliferation [46] and independent of the maturation process the FGF signalling pathway is regulating myelin growth [47]. The regulated expression of members of the FGF family throughout the maturation of oligodendrocytes presents an opportunity for therapeutic intervention that has not yet been explored.

### 2.2. Microglia

Microglia participate in the removal of myelin debris and produce growth factors, including the glial cell line derived neurotrophic factor [48], that are favourable for neurite growth and regeneration [49]. Microglia express FGF2 and induce increased FGF2 expression in neurons which results in neuroprotection [50]. To our knowledge there is no therapeutic research done on microglia with respect to the FGF superfamily. However, this could be a potential route for interference.

### 2.3. Astrocytes

Astrocytes express FGF2 which plays a role in their differentiation [51, 52] and proliferation [53]. Furthermore, it has been shown in vivo that astrocytes express FGFR1 mRNA and protein [54–56], as well as FGFR2 mRNA [57]. Further, it has been shown in vitro that they also express FGFR3, however, at a lower level than FGFR1 and 2 [58]. FGFR3 expression by astrocytes has also been found in vivo and signalling via this receptor has been associated with repression of GFAP expression [59]. Astrocytes transform after injury into reactive astrocytes. Reactive astrocytes express and secrete higher levels of FGF2 after SCI, which in turn promotes proliferation and survival of OPCs [60–63]. The effect or reactive astrocytes on motoneurons is diverse. For instance, they induce via a p75NTR dependent mechanism oxidative stress in motoneurons and subsequent death of the same [64]. Simultaneously, oxidative stress in motoneurons results in FGF1 release from motoneurons which enhances activation of astrocytes [65] which might be leading to increased scar formation. Furthermore, astrocytes have been shown in vitro to be attracted by FGF2 [66]. Since the amount and reactivity of astrocytes have been proven essential for the quality of the scar that defines whether it is detrimental or beneficial, we believe that also astrocytes could be a target of FGF induced therapy.

## 3. Expression of FGF Ligands in the Intact and Injured Spinal Cord

### 3.1. Expression of FGF1 and FGF2 before and after Injury

All cell types of the nervous system express FGF ligands and receptors at a basal level; however, SCI results in changes of their expression pattern which allows together with other factors a limited degree of spontaneous regeneration after SCI. Several studies confirmed that expression of FGF1 and FGF2 in the injured cord is upregulated at the mRNA and protein level: FGF2, but not FGF1 mRNA, is upregulated after an incomplete thoracic contusion in spinal cord tissue including the lesion site [78]. Furthermore, FGF2 protein has been found upregulated after contusion in glial cells, along blood vessels and surrounding neurons [79]. Koshinaga et al. investigated in detail the distinct increase of FGF1 and FGF2 in different anatomical structures and at different time points after a photochemically induced complete destruction of the dorsal columns of rats at T8. They propose that FGF1 and 2 have distinct roles after SCI since they show very differential cellular, temporal, and spatial expression following after destruction of the dorsal columns [80]. In the uninjured spinal cord FGF1 is expressed in the cytoplasm of ventral motor neurons and sensory fibres in the dorsal columns. FGF2 is expressed in astrocytic nuclei and the cytoplasm of few neurons of the grey matter. After the lesion they found a distinct cellular, temporal, and spatial expression of FGF1 and FGF2, which suggests separate roles for them in response to SCI. They investigated the expression level of FGF1 two and five days after injury at the lesion site, above the lesion (T4-5) and below the lesion (L1-2). They found that two days after injury FGF1 protein was upregulated in the ventral motor neurons and intermediate grey matter at the lesion site. Furthermore, FGF1 protein was expressed in the (spared) fasciculus cuneatus at T4-5, but not in the (lesioned) fasciculus gracilis at the same level (note that the lesion was at thoracic vertebral levels T8). However, at L1-2 FGF1 protein expression was increased in the fasciculus gracilis, which suggests that normally FGF1 is anterogradely transported. This was assessed by immunohistochemistry against FGF1. The authors do not co-stain with other markers, but draw their conclusions from the anatomy and the morphology of the stained structures. FGF2 protein in contrast was unchanged 2 days after surgery but upregulated five days after the lesion in the nucleus and cytoplasm of reactive astrocytes at the edge of the cystic cavity and in the dorsal columns at T4-5. In summary, there is very specific upregulation of FGF1 and 2 in distinct anatomical structures and cells.

### 3.2. FGF22 Signalling after Injury

It has been shown that one mechanism of spontaneous regeneration after SCI is formation of new intraspinal circuits in order to circumvent the lesion site [1, 81]. New formation of circuits requires newly formed CST collaterals to enter the cervical grey matter and formation of synapses to new targets, for example, long propriospinal neurons [1]. The group of Florence Bareyre has shown that FGF22 signalling via FGFR1 and FGFR2 is essential for the formation of these new synapses [82]. They showed that FGF22 is expressed in spinal interneurons, including a large proportion of long propriospinal neurons, and that FGFR1 and 2 are expressed in the CST of mice. They found that ablation of either FGF22 or one or both of the receptors in conditional knockout mice leads to decreased synapse formation between newly formed CST collaterals and propriospinal relay neurons after thoracic dorsal hemisection. Further, this lead to inhibited functional recovery after the lesion proven by showing that the genetically altered animals performed more mistakes with their hindlimbs on the horizontal ladder and mice had an abnormal angle between the hindlimb paws and body axis as measured on the Catwalk [82]. There is further evidence that FGF22 has a role in synapse formation in the CNS [83].

## 4. Therapeutic Approaches Targeting the FGFR Pathway

For functional regeneration axotomised axons have to grow around or through the glial scar and form functional synapses distal to the lesion site. One line of therapeutic research aims at promoting axon regeneration by manipulating the FGFR signalling pathway.

### 4.1. Therapies Delivering FGF1 to the Injury Site

The study of Cheng et al. set out to ensure that the injury site is supplemented with FGF1 for a prolonged time directly after a complete transection injury at T8 in rats [67]. To achieve this, they bridged the gap resulting from the removal of 5 mm of the spinal cord by using 18 fine segments of autologous peripheral nerve implant with fibrin glue containing FGF1. The fibrin glue was designed to provide slow release of FGF1 [84]. To evade oligodendroglial proteins that inhibit regeneration they aimed to transplant the nerve segments in a way to link from the nonpermissive white matter to the permissive grey matter, thereby rerouting descending motor and ascending sensory pathways. However, there is no direct evidence that they successfully achieved this. To stabilise the lesion site the authors applied fibrin based tissue glue and fixated the vertebrate column by dorsiflexion wiring [67]. The study has been extensively controlled by four control groups: (1) transection only, (2) transection with removal of 5 mm of the spinal cord, (3) transection with grafting but routing the fibres through white matter only, and (4) transection with grafting but omission of FGF1. Two independent blinded observers assessed the combined behavioural score and the open-field walking score. The authors found 3 weeks after injury and persistent throughout the 12 months of monitoring that the experimental group of transected, grafted, and FGF1 treated rats had flexion at the hips and knees and dorsiflexion of the ankle, partial body weight support, absence of toe dragging, and contact placing while the control rats (all four groups) had fully extended, externally rotated hindlimbs. The observed contact placing in the experimental group is indicative of CST regeneration. To test this, they performed retro- and anterograde tracing experiments. The authors state that axons regenerated into the graft and beyond into the distal host tissue; however, they only show the tracing in one animal from the sensorimotor cortex to the lumbar section of the spinal cord. The pictures are difficult to interpret without a negative control; therefore, the evidence for regeneration beyond the lesion site is not entirely convincing. Interestingly, they state that there are cavities surrounded by glial fibrillary acidic protein- (GFAP-) rich regions between the spinal cord stumps in the experimental group. The group of Jerry Silver used several years later the same approach of injury and nerve grafting that Cheng et al. used and investigated the rescue of bladder function. This study is discussed in detail below. However, we would like to mention here that Jerry Silver's group found evidence that axons regenerate mostly at these GFAP-rich sites from the host into the graft [76]. The group of Cheng later investigated a possible mechanism of the increased regeneration of peripheral nerve graft and FGF1 treated animals. They found that a peripheral nerve graft alone increases the level of CSPGs at the junction of graft and host tissue and in the degenerative area. However, addition of FGF1 to the peripheral nerve graft or FGF1 treatment alone reduced the level of CSPGs [85]: However, they did not show if the differential level of CSPGs actually has an effect on the regenerating axons. In summary, these data indicate that FGF1 may modify the host-graft interface to make it more permissive to axon regeneration.

Lee et al. 2002 used the same approach as Cheng et al. and corroborated their earlier findings. Comparable to the original study they observed an increase in the BBB score from one to seven comparing the transected, grafted, and FGF1 treated experimental group to four control groups (laminectomy only, transection only, FGF1 releasing matrix only, and transection plus nerve graft and fibrin matrix without FGF1) [68]. Furthermore, they reproduced the observation that there is partial recovery of hind-paw contact-placing reflexes in the experimental group. However, in addition to Cheng et al. they performed electrophysiology and showed that not only locomotor function was partially restored, but also ascending sensory pathways. They measured somatosensory evoked potentials (SSEPs) in the sensory cortex after stimulation of the sciatic nerves at very high intensity (20 mA). The authors found in the injured rats four month after injury treated with FGF1 and peripheral nerve grafts similar latencies in SSEP as in sham operated rats, but lower amplitudes. This effect was lost after a retransection of the spinal cord. They state that this indicates that there were motor and sensory fibres that grew through the bridge to form synapses on distal pathways. However, nonspecific effects of high intensity stimulation (e.g., stimulus spread) cannot be ruled out. Importantly, in this study there was a control group that received the nerve graft and fibrin matrix but no FGF1. This group did not show improved regeneration compared to the other control groups, which suggests that it is the combination of nerve graft and FGF1 treatment which is needed to achieve regeneration and reconnection. Together, Cheng et al. and Lee et al. present evidence that the combination of nerve graft bridging and FGF1 releasing fibrin matrix results in regeneration of some motor and sensory fibres across the transection injury.

In contrast to peripheral nerve grafts Guest and colleagues used human Schwann cell grafts supplemented with FGF1 [69]. They performed a mid-thoracic spinal cord transection in adult athymic nude rats, a xenograft tolerant strain. At the time of injury, they bridged the gap with a human Schwann cell graft with or without FGF1-containing fibrin glue placed at the injury-graft boundary. Ten days after the injury and the grafting the dorsal surface of the guidance channel was incised and a small segment was removed to place a fresh aliquot of 10 *μ*L glue containing FGF1 and reseal the opening. Thirty-five days after injury and treatment they found that grafted and FGF1 fibrin glue treated animals showed CST axon regeneration into the graft while control animals did not do so. Furthermore, they showed that the maximum termination density is closer to the rostral host-graft interface and that there is less axonal die-backmeasured by the longitudinal spread of bulbous end terminals. The authors offer two mechanistic hypotheses: (1) intrinsic: FGF1 is retrogradely transported in the CST and improves the regenerative capacity of the CST. This might be feasible since it has been shown that FGF1 is both anterogradely [80] and retrogradely [86] transported in ascending sensory fibers and enhances PNS regeneration [87]; (2) extrinsic: the FGF1 fibrin glue alters the host-graft boundary in a way that makes the local environment growth permissive. In favour for this hypothesis is the fact that FGF1 alters the behaviour of glial cells [69] and that reactive astrocytes have been thought of expressing high levels of FGF1 receptors [56, 57]. In summary, Guest et al. report that human Schwann cell grafts supplemented with FGF1 after complete transection result in ingrowth into the graft of regenerating axons; however, there was no evidence that axons also grew into the distal host tissue.

Together these studies show that addition of FGF1 to the lesion site after a complete transection and grafting of peripheral nerve segments or human Schwann cells enhances regeneration of axons to a limited extent.

A summary for therapeutic approaches using FGF1 delievery can be found in Table 1.

### 4.2. Therapies Enhancing FGF2 Concentration in the Injured Spinal Cord

There was another approach using Schwann cell grafts. Rat Schwann cell grafts have been combined with FGF2 supplementation after a complete transection at T9/T10 with removal of 4 mm of spinal cord in rats [70]. However, this study showed a less promising outcome than the previously discussed study of Guest et al. In Meijs' study, the only difference in outcome between FGF2 supplemented Schwann cell grafts and control Schwann cell grafts was the increased survival of NeuN positive cells in the posterior cord. There was no difference in the amount of white matter sparing, axonal ingrowth into the graft, the ratio of unmyelinated to myelinated axons and BBB score between the experimental and the control group. However, the authors found higher intensity of CSPG and GFAP staining. Upregulated levels of GFAP positive, reactive astrocytes that secrete CSPGs might explain the lack of regeneration since they may inhibit any axon regeneration across the host-graft boundary. However, this theory offered by the authors is in contrast to later findings that show that longitudinally aligned astrocytes may serve as substrate for growth at the injury site [22, 76]. It is quite intriguing that Schwann cell grafts with FGF1 have such a positive outcome and Schwann cell grafts with FGF2 do not. However, there are some critical differences between the two studies. The FGF1 study was done with human Schwann cell grafts in nude rats and the FGF2 study was done with rat Schwann cell grafts in Fisher rats. In the FGF1 study they placed the FGF1 at the two graft-host tissue interfaces while in the FGF2 study they mixed the Schwann cells with the FGF2. Also, in the FGF1 study they added 10 days after the injury fresh fibrin containing FGF1into the channel. This was not done in the FGF2 study. These differences may account for the different outcomes.

Sasha Rabchevsky pursued the effect of FGF2 upon regeneration by using osmotic minipumps while working in the group of Stephen Scheff [71, 72]. After a moderate contusion injury at T10 in rats they implanted two osmotic minipumps per animal delivering FGF2 into the lateral ventricle and the lumbar thecal sac between 30 minutes to one week after injury. They reported that this treatment with FGF2 resulted in an improved BBB score of 15/16 four to six weeks after injury compared to control animals reaching 12/13 only [71]. Furthermore, they found that there was more tissue sparing in the FGF2 treated animals compared to control animals. They conclude that the observed effects are due to the neuroprotective effect of FGF2, which has been reported previously [88–96]. They later confirmed these results in a more severe injury model [72]. In this study they observed improvement in the BBB score from 7–10 to 10–13. However, they did not observe improved tissue sparing or a difference in number of astrocytes and microglia when compared to control animals. Therefore, the authors conclude that there is an as yet undefined mechanism of FGF2 contributing to enhanced functional recover.

Kasai et al. injected FGF2 directly after a complete transection at T10 into the surrounding spinal cord tissue of rats [73]. They chose this approach over a slow releasing method since they believe that the storage of FGF2 in the extracellular matrix after injection should be sufficient. Gonzalez et al. showed that FGF2 could be detected in the rat brain four and seven days after FGF2 injection [97]. During the six weeks in which they assessed the rats behaviourally they found an increase in the BBB scale of the treated rats versus untreated rats. While the control group did not improve on the BBB scale at all and remained at a score of zero, the FGF2 injected rats reached on average a score of nearly six, moving two of their hindlimb joints six weeks after complete transection. They also performed tracing experiments with fluorogold (FG) in the spinal cord 5 mm caudal to the injury site. The animals were injected with FG 6 weeks after injury and euthanised 3 days after FG injection. They report positive neural cell bodies in the sensorimotor cortex and red nucleus after FG trancing. Even though the number of FG positive cell bodies is significantly less than in sham operated animals, the authors believe that this indicates regeneration of CST and/or rubrospinal pathways. The authors also show evidence for the presence of FGF2 induced fibronectin positive cells that fill the cystic cavities and allow neurons to grow into the injury site. However, the authors do not show any immunohistochemistry that would show direct evidence of axon growth through the lesion site. The locomotor improvement observed in the BBB does not necessarily indicate regeneration of axons but could also result from changes in the lumbar central pattern generators (CPGs).

Another group investigated the effect of FGF2 on the immune reaction in mice [74]. They injected FGF2 subcutaneously during two weeks after a T12 lateral hemisection. Comparable to the already discussed studies they found a locomotor improvement of the animals treated with FGF2 as assessed by the grid test and the mouse-modified open field test for mice (mBBB). Further, they discovered that the mRNA level of the proinflammatory factor TNF*α* is reduced at the lesion site leading to decreased microglia and macrophage activation. While they did not observe lower total numbers of astrocytes they found fewer activated astrocytes which they believe led to less abundant CSPG as they observed it. Furthermore, they showed that FGF2 mediated astrocyte bipolar morphology, which leads to astrocytic bridges through the lesion site onto which growing nerve fibres can follow, as has been shown earlier in zebrafish [98] and rats [76]. This study shows that FGF2 treatment also affects the immune reaction after SCI. This in turn opens the question if the studies discussed above elicited also a differential immune response depending on the addition of FGF2 or not. Also, the question remains whether administering FGF2 via injection, either into the spinal cord or subcutaneously, raises a different immune response then applying a fibrin matrix that contains the ligand. Certainly it seems to be more feasible to inject FGF2 subcutaneously rather than intraspinally.

The group of de Oliveira Costa used gel foam containing sciatic nerve fragments plus FGF2 or PBS to bridge a T10 complete transection in rats [75]. They found that there is a therapeutic effect of the sciatic nerve fragment transplantation itself that could be increased only in some behaviour tests by the addition of FGF2. They showed that rats that were transplanted with nerve fragments reached score four and rats that only had the gel foam implanted reached score one on the BBB scale eight weeks after injury. Furthermore, animals that received the nerve graft containing FGF2 reached score six in the BBB test. However, in the combined behaviour score (CBS) test there was no therapeutic effect between the group with the nerve transplant only and the group with the nerve transplant plus FGF2, and both groups showed a significant effect compared to gel foam only transplanted animals. The studies discussed here show that FGF2 has the potential to promote growth of axons. They also show that FGF2 alters the immune response which might have an influence on the axon regeneration. We believe that these studies also exemplify that the timing and route of application is important, as made apparent by the two studies with Schwann cell grafts that had very differential outcomes.

We have so far discussed that FGF1 and FGF2 induce regeneration of axons when applied either alone or in combination with a nerve- or cellular bridges. Next, we will discuss combination therapies that either combine intrinsic and extrinsic effects or different effects of FGF ligands.

A summary of FGF2 dependent therapies can be found in Table 2.

### 4.3. Combination Therapy

The functional effects that are achieved by the presented strategies are mostly positive; however they are small and it has to be the aim to get even better effects that might be translatable. One approach should be to combine intrinsic neuronal effects with extrinsic effects. The groups of Lee and Silver performed such a combinatorial study [76] and this will be discussed in more detail here. They used the approach developed by Cheng et al. However, they included an experimental group that received Chondrointinase ABC (ChABC) in addition to peripheral nerve grafts (PNGs) and FGF1 (PNGs + FGF1 + ChABC) [76]. ChABC is a bacterial enzyme that cleaves the inhibitory sugar chains of the CSPGs [99]. The authors bridged the injury gap with 18 intercostal nerve segments to produce PNGs, soaked the PNGs with ChABC, and covered them with an FGF1-laden fibrin matrix. Furthermore, they injected ChABC into the interface of graft and host. This study focused solely on the regeneration of bladder function. They showed that the transection + PNG + FGF1+ ChABC treated animals (experimental group) had significantly better bladder function than all five control groups (laminectomy only, transection only, transection + PNG, transection + FGF1 + ChABC, transection + PNG + FGF1, transection + PNG + ChABC). Importantly, they found that transection + PNG + FGF1 and transection + PNG + ChABC groups had significantly better bladder functions than the other control groups, but not as good as the experimental group. In addition they showed that serotonin (5-HT) and tyrosine hydroxylase (TH) positive fibers, both important for urination (micturition), had extended into the bridge, across the caudal PNG/spinal cord interface and well into the caudal cord [76]. This phenomenon was also present in the PNG + FGF1 treated animals, but again to a lesser extent than the PNG + FGF1 + ChABC animals. Injections of a retrograde tracer below the bridge in the L4 spinal cord segment showed that in both groups fibres of the D-region (important for micturition), raphe magnus nuclei, reticular formation, and the cervical spinal cord extended into the host tissue caudal to the bridge. However, PNG + FGF1 + ChABC treated animals showed more labelled cells and in more neural populations than did the PNG + FGF1 group. Furthermore, as mentioned before, they showed that astrocytes aligned at the interface of PNG and host and they had indications that it was at these sites of astrocyte alignment where axons entered the distal cord [76]. Since this study has been so extensively controlled with five control groups they were able to show that the combination of carefully chosen factors can increase the beneficial effect of therapies.

A different approach of exploring synergism between treatments was taken by ourselves. We hoped to combine the different effects that the various FGF ligands had by overexpressing their common receptor, the FGFR1 [77]. We did that in a rat model of unilateral pyramidotomy. We injected an AAV serotype 1 overexpressing either the FGFR1 or mCherry as control one week prior to the injury into the sensorimotor cortex supplying the CST that will be left intact. After injuring the contralateral CST with a pyramidotomy we assessed the animals behaviourally by the horizontal ladder with unevenly spaced rungs and the Montoya staircase test. In addition, we investigated sprouting of the intact contralateral CST fibres that overexpressed FGFR1 or mCherry, over the spinal midline in the cervical cord. However, in all our tests we did not find a difference between control and FGFR1 overexpressing animals. Furthermore, we investigated overexpression of FGFR1 in vitro in cerebellar granule neurons and we found that overexpression of FGFR1 results in decreased neurite outgrowth compared to control cells overexpressing GFP. We hypothesis that this effect is due to the sequestering of adaptor proteins, such as FRS2, away from other proregenerative pathways, such as NGF-TRKA signalling. It has been shown by others that overexpressed FGFR1 sequestering adaptor proteins away from other proneurite outgrowth pathways [100] and that FGF2 can have an inhibitory effect on neurite outgrowth in cerebellar neurons growing on monolayers of cortical astrocytes [101]. This last study exemplifies that combining unfavourable or antagonistic factors can be counter-productive.

Several groups have shown that combination therapies involving grafts bridging the injury site and addition of FGF1 or 2 and possibly other factors can improve outcome even after severe spinal cord injury.

Combination therapies are summarized in Table 3.

## 5. Conclusion

Most FGFR signalling related therapies discussed in this review show potential to improve plasticity and result in enhanced axon growth and improvement of motor related functions. However, the achieved improvements are small and need to be enhanced. This might be achieved by using combined therapies as we have illustrated in this review with the work of Lee et al. It is evident that combination of proregenerative strategies can increase the observed motor improvements, but again the benefits are limited and often only confined to specific outcomes and not a general phenomenon. We therefore believe that there is a need to understand the distinct effects better to combine therapies in a better temporal and spatial manner.

The disadvantages of most of the described studies is that they use highly invasive FGF application techniques, often involving autologous transplantation that results in multiple invasive procedures. We believe that the focus should shift towards more translatable techniques; however, it is clear that this is not trivial for a ligand that should be delivered very locally in high concentrations over a period of time. We, ourselves, have tried one alternative approach by injecting into the motor cortex adeno associated viruses (AAVs), a delivery method with the advantage of being already used in humans, however, still rather invasive [77]. Unfortunately, this treatment showed no beneficial effects. A possible outcome improvement strategy for this and other studies might be the combined overexpression of the FGF receptor and ligand as opposed to our study which only used overexpression of the FGFR1. Another very minimally invasive approach has been chosen by Goldshmit et al. which performed a successful study with subcutaneous FGF injections [74]. This benefits from the fact that the blood-brain-barrier (BBB) is open due to the injury and the injected FGF can reach the injury site. However, it is not clear if the opening of the BBB correlates with the optimal FGF treating window and this strategy will not allow a more prolonged FGF treatment. A possible disadvantage of systemic application of FGF might be unexpected adverse side effects. This has so far not been investigated in context with spinal cord injury treatment. The invasive, but local delivery of FGF ligands, has so far not led to major adverse side effects; however, a more global treatment with FGF ligands could result in a wide range of problems, since the FGFR pathway has so many diverse functions in the whole organism and plays an important role in the progression of cancer.

All these studies used different FGF delivery methods (though most of them use slow-release matrices) and different time regimes and injury models. This makes it difficult to compare them directly. It has been discussed before that different studies will lead to different outcomes mainly due to the experimental set-up rather than true regenerative potentials [102].

Most studies focused on improving the motor outcome after treatment and only very few report observations on regeneration of sensory fibres. It would therefore, for the future, be interesting to shed some light on the regeneration of sensory fibres after FGF treatment since both, sensory and motor fibres, need to regenerate for clinical improvement.

## Figures and Tables

**Table 1:** Different strategies to promote regeneration using FGF1.

Species	Model	Therapeutic intervention	Outcome	Control	Ref
Rat	Complete transection at T8. Removal of 5 mm of SC.	Autologous peripheral nerve implant with glue containing FGF1. Routing of regenerative pathway from white matter to grey matter.	Improvement on open field walking score from 0.5 to 3. Axon regeneration beyond the graft. Fewer GFAP poor holes between the stumps.	Transection only. Cord removal only. White matter-to-white bridging. Omission of FGF1.	[67]

Rat	Complete transection at T8. Removal of 5 mm of SC.	Autologous peripheral nerve implant with glue containing FGF1.	Improvement on BBB from 1 to 7. Partial restoration of sensory function.	Transection only, laminectomy only. Transection and peripheral nerve graft and matrix fibrin matrix without FGF1. Transection and FGF1 fibrin matrix.	[68]

Nude rat	Mid-thoracic SC transection.	Human Schwann cell graft with FGF1 fibrin glue at the injury-graft boundary and delayed FGF1 fibrin glue at the dorsal surface of the guidance channel.	Axon regeneration into the graft. Maximum termination density closer the host-graft interface. Less axonal die-back.	Schwann cell graft without FGF1.	[69]

**Table 2:** Different strategies to promote regeneration or neuroprotection using FGF2.

Species	Model	Therapeutic intervention	Outcome	Control	Ref
Rat	Complete transection at T9/T10. Removal of 4 mm of SC	Rat Schwann cell grafts with FGF2 fibrin glue	Increased survival of NeuN positive cells	Schwann cell graft without FGF2	[70]

Rat	Moderate contusion injury at T10	Osmotic minipump in the lateral ventricle and lumbar thecae sac releasing FGF2 30 min after injury for 1 week	Improvement of BBB score from 12/13 to 15/16. More tissue sparing	Osmotic minipump releasing bovine serum albumin	[71]

Rat	Severe contusion injury at T10	Osmotic minipump in the lateral ventricle and lumbar thecae sac releasing FGF2 30 min after injury for 1 week	Improvement of BBB score from 7–10 to 10–13	Osmotic minipump releasing bovine serum albumin	[72]

Rat	Complete Transection at T10	Direct injection of FGF2 into the surrounding tissue of injury	Improvement of BBB score from 0 to 6	Injection of vehicle	[73]

Mouse	T12 hemisection	Subcutaneous injection of FGF2 during 2 weeks	Better performance on the grid test and mBBB. Reduced level of TNF*α* leading to decreased microglia and macrophage activation	Injection of vehicle	[74]

Rat	T10 complete transection	Bridging with gel foam containing sciatic nerve fragments and FGF2	Improvement in BBB from 1 to 6	Bridging with gel foam containing sciatic nerve fragments and PBS	[75]

**Table 3:** Combination therapy.

Species	Model	Therapeutic intervention	Outcome	Control	Ref
Rat	T10 complete transection	18 intercostal segments (peripheral nerve autografts (PNGs)) soaked with ChABC and covered by FGF1-laden fibrin matrix, plus ChABC injection in the interface of graft and host.	Significantly better bladder function. Regeneration of 5-HT and TH positive fibres into and beyond the graft	Laminectomy only. Transection only. Transection plus PNG. Transection plus FGF1 and ChABC. Transection plus PNG and ChABC. Transection plus PNG	[76]

Rat	Unilateral pyramidotomy	Overexpression of FGFR1 via AAV1 injections into the unlesioned CST	No difference between FGFR1 overexpressing animals and control animals	Injection of AAV1 overexpressing mCherry	[77]
